# Effectiveness and safety of integrase strand transfer inhibitors in Spain: a prospective real-world study

**DOI:** 10.3389/fcimb.2023.1187999

**Published:** 2023-06-26

**Authors:** José Ramón Santos, Maria Casadellà, Marc Noguera-Julian, Rafael Micán-Rivera, Pere Domingo, Antonio Antela, Joaquin Portilla, Jesús Sanz, Marta Montero-Alonso, Jordi Navarro, Mar Masiá, Nieves Valcarce-Pardeiro, Antonio Ocampo, Laura Pérez-Martínez, Coral García-Vallecillos, María Jesús Vivancos, Arkaitz Imaz, José Antonio Iribarren, José Hernández-Quero, Judit Villar-García, Pilar Barrufet, Roger Paredes, INSTINCT study group, Mariona Perera

**Affiliations:** ^1^ Fight Infections Foundation, Service of Infectious Diseases, Hospital Universitari Germans Trias i Pujol, Badalona, Spain; ^2^ IrsiCaixa AIDS Research Institute, Badalona, Spain; ^3^ HIV Unit, University Hospital La Paz, Madrid, Spain; ^4^ Infectious Diseases Unit, Hospital de la Santa Creu i Sant Pau, Barcelona, Spain; ^5^ Infectious Diseases Unit, Santiago de Compostela Clinical University Hospital, Santiago de Compostela, Spain; ^6^ Department of Internal Medicine, Hospital General Universitario Dr. Balmis de Alicante, Alicante, Spain; ^7^ Department of Infectious Diseases, University Hospital de La Princesa, Madrid, Spain; ^8^ Infectious Diseases Unit, La Fe University and Polytechnic Hospital, Valencia, Spain; ^9^ Infectious Diseases Department, Hospital Universitari Vall d’Hebron, Barcelona, Spain; ^10^ Infectious Diseases Unit, Elche University General Hospital, Elche, Spain; ^11^ Centro de Investigaciones Biomédicas en Red de Enfermedades Infecciosas (CIBERINFEC), Instituto de Salud Carlos III, Madrid, Spain; ^12^ Infectious Diseases Unit, Complexo Hospitalario Universitario de Ferrol (CHUF), Ferrol, Spain; ^13^ HIV Unit, Hospital Álvaro Cunqueiro, Vigo, Spain; ^14^ Department of Infectious Diseases, Biomedical Research Center of La Rioja (CIBIR), Logroño, Spain; ^15^ Infectious Diseases Unit, University Hospital Virgen de las Nieves, Granada, Spain; ^16^ Department of Infectious Diseases and Instituto Ramón y Cajal de Investigación Sanitaria (IRYCIS), Ramón y Cajal Hospital, Madrid, Spain; ^17^ HIV and STI Unit, Infectious Diseases Department, Bellvitge University Hospital, Bellvitge Biomedical Research Institute (IDIBELL), Hospitalet de Llobregat, Spain; ^18^ Department of Infectious Diseases, Donostia University Hospital, Instituto de Investigación Sanitaria BioDonostia, Universidad del País Vasco, San Sebastián, Spain; ^19^ Service of Infectious Diseases, University Hospital San Cecilio, Granada, Spain; ^20^ Infectious Diseases Department, Hospital del Mar - Institut Hospital del Mar d'Investigacions Mèdiques (IMIM), Barcelona, Spain; ^21^ Infectious Diseases Unit, Mataró Hospital, Mataró, Spain

**Keywords:** HIV, integrase strand transfer inhibitors (INSTI), real-world study, raltegravir, elvitegravir, dolutegravir

## Abstract

**Introduction:**

Second-generation integrase strand transfer inhibitors (INSTIs) are preferred treatment options worldwide, and dolutegravir (DTG) is the treatment of choice in resource-limited settings. Nevertheless, in some resource-limited settings, these drugs are not always available. An analysis of the experience with the use of INSTIs in unselected adults living with HIV may be of help to make therapeutic decisions when second-generation INSTIs are not available. This study aimed to evaluate the real-life effectiveness and safety of dolutegravir (DTG), elvitegravir/cobicistat (EVG/c), and raltegravir (RAL) in a large Spanish cohort of HIV-1-infected patients.

**Methods:**

Real-world study of adults living with HIV who initiated integrase INSTIs DTG, EVG/c, and RAL-based regimens in three settings (ART-naïve patients, ART-switching, and ART-salvage patients). The primary endpoint was the median time to treatment discontinuation after INSTI-based regimen initiation. Proportion of patients experiencing virological failure (VF) (defined as two consecutive viral loads (VL) ≥200 copies/mL at 24 weeks or as a single determination of VL ≥1,000 copies/mL while receiving DTG, EVG/c or RAL, and at least 3 months after INSTI initiation) and time to VF were also evaluated.

**Results:**

Virological effectiveness of EVG/c- and RAL-based regimens was similar to that of DTG when given as first-line and salvage therapy. Treatment switching for reasons other than virological failure was more frequent in subjects receiving EVG/c and, in particular, RAL. Naïve patients with CD4+ nadir <100 cells/μL were more likely to develop VF, particularly if they initiated RAL or EVG/c. In the ART switching population, initiation of RAL and EVG/c was associated with both VF and INSTI discontinuation. There were no differences in the time to VF and INSTI discontinuation between DTG, EVG/c and RAL. Immunological parameters improved in the three groups and for the three drugs assessed. Safety and tolerability were consistent with expected safety profiles.

**Discussion:**

Whereas second-generation INSTIs are preferred treatment options worldwide, and DTG is one of the treatment of choices in resource-limited settings, first-generation INSTIs may still provide high virological and immunological effectiveness when DTG is not available.

## Introduction

Antiretroviral therapy (ART) coverage for people living with HIV had reached 28 million people as of mid-2021 (UNAIDS, 2021). Consistent with ART optimizing principles, availability of fixed-dose combinations, better tolerability, and the low rate of transmitted resistance mutations in the integrase, current WHO recommendations consider the integrase strand transfer inhibitor (INSTI) dolutegravir (DTG) as the preferred drug in first- and second-line ART (World Health Organization, 2021). Currently, DTG and bictegravir (BIC) are the most commonly used INSTIs for either initiating, rescuing, or switching ART (Vitoria et al., 2018; World Health Organization, 2021). However, alternative ART regimens based on other INSTIs are extensively used, especially when there is limited availability of DTG and BIC or when drug-drug interactions must be considered. Therefore, in adults living with HIV with limited treatment options, raltegravir (RAL) and elvitegravir (EVG/c) plus optimized background therapies still play a role in ART.

The effectiveness of therapy in daily clinical practice usually differs from what is observed in clinical trials. Factors like different nucleoside reverse-transcriptase inhibitor (NRTI) backbones, the impact of polymorphic mutations on INSTIs (Orrell et al., 2017; WHO, 2021), gender, and the diversity of socioeconomic and demographic characteristics can influence the effectiveness of ART, including INSTIs. In addition, most ART challenges in low-income countries are mainly rooted, among other factors, in HIV drug resistance and the scarcity of alternative treatment options, more so in adults living with HIV who failed salvage therapy (Dorward and Hamers, 2019; Ndashimye and Arts, 2019; World Health Organization, 2021). Therefore, it is still important to analyse the experience with the use of INSTIs in unselected HIV-infected patients (Freemantle et al., 2005; López-Martínez et al., 2012; Bérard, 2021) and their effectiveness to evaluate the real-life performance of these treatments.

This study aimed to evaluate the real-life effectiveness and safety of DTG-, EVG/c-, and RAL-based regimens in a large Spanish cohort of HIV-1-infected patients before BIC became commercially available.

## Material and methods

### Study design

The INSTINCT study is a multicentre, prospective, observational 96-week study including a cohort of HIV-positive patients who initiated a RAL-, EVG/c-, or DTG- based regimen between 1 April 2015 and 5 October 2016 in 19 Spanish HIV care centres. The study included ART-naïve, ART-switching, and ART-salvage patients initiating an INSTI for the first time.

Adults over 18 years old with confirmed HIV-1 infection starting antiretroviral treatment with RAL, EVG/c or DTG for any reason at the time of study initiation, were enrolled. Two-drug regimen initiation was allowed. Exclusion criteria included previous INSTI-based regimen initiation as part of chemoprophylaxis (post- and pre-exposure). BIC was not included in this study because it was not commercially available when it started.

Patients who fulfilled inclusion criteria were allocated to one of the following groups: (a) ART-naïve, i.e., previously untreated patients at the time of INSTI initiation; (b) ART-switching, i.e., ART-experienced patients initiating the new INSTI with viral load (VL) ≤500 copies/mL; and (c) ART-salvage, i.e., ART-experienced patients initiating the new INSTI with VL >500 copies/mL due to virological failure (VF) and patients who were on voluntary ART interruption.

### Data and assessments

Data were collected at the date of the INSTI initiation (baseline) and every 24 weeks up to 96 weeks, according to the study protocol. In addition to demographic characteristics, other variables recorded were: HIV-1 RNA levels, CD4+ T-cell counts, CD4+ nadir, haematological and biochemical parameters, previous ART history, ART adherence (calculated as no. of taken capsules/no. of prescribed capsules x100), reasons for initiating INSTI-based regimens and ART following INSTI discontinuation, and previous genotypic test results (i.e., before INSTI initiation, when available, and at VF). Adverse events (AEs) were classified according to MedDRA (version 25.0) standard terminology.

The primary endpoint was the median time to treatment discontinuation after INSTI-based regimen initiation. In addition, the proportion of patients experiencing VF and time to VF for each regimen were also evaluated. VF was defined as two consecutive VL ≥200 copies/mL at 24 weeks, or as a single determination of VL ≥1,000 copies/mL while receiving RAL, EVG/c or DTG, and at least 3 months after INSTI initiation. The first date of confirmed VL ≥200 copies/mL was used to define the time at which VF had occurred. Secondary endpoints were time to the composite endpoint of VF or INSTI discontinuation due to adverse events, factors associated with VF and INSTI-based regimens discontinuation due to any reason, development of INSTI resistance in cases of VF, and changes in CD4+ T-cell counts. A restrictive secondary effectiveness analysis was performed defining VF as two consecutive VL ≥50 copies/mL at 24 weeks. In addition, safety variables, including AEs leading to INSTI discontinuation, AIDS progression, death for any reason, and changes in haematological and biochemical parameters in patients receiving INSTI regimens were evaluated.

### Statistical analysis

Continuous variables were summarized as the mean and standard deviation (SD) and the median and interquartile range (IQR), as required, and categorical variables were described as frequencies and percentages. Variables were transformed and/or results stratified according to data distribution. Differences were compared using the chi-squared test for categorical variables and nonparametric tests for continuous variables.

Given the real-world nature of this study, no formal sample size calculation was performed. A two-year recruitment period was planned, resulting in an estimated study sample of 1,000 patients (500 patients/year).

The Kaplan–Meier survival analysis was used to determine the median time to VF and the proportion of patients who experienced VF within the 96 weeks from INSTI-based regimen initiation. Treatments were compared using the log-rank test. A Cox proportional hazard regression model was used to identify factors associated with each of the three considered outcomes. Multivariable models were fitted using 0.05 as the significance level in univariable analysis for a covariate to be included in the final multivariable model. Hazard ratios (HRs) and 95% confidence intervals (95% CIs) were calculated. The relationship between the final set of included covariates was assessed, and those showing collinearity were removed from the model. Effectiveness was evaluated with an intention-to-treat (ITT) analysis ignoring treatment switches of INSTI (genuine ITT analysis). Sensitivity analyses were performed counting any INSTI discontinuation [ITT: switching = failure (S=F)] and any loss to follow-up [ITT: missing = failure (M=F)] as failures. All statistical analyses were performed using R software (version 4.0).

### Ethics

The study was conducted in accordance with the Helsinki Declaration and the local Personal Data Protection Law (LOPD 15/1999). The study protocol was approved by the local or regional (autonomous community) ethics committee of each participating centre, and all patients gave their written informed consent prior to participation (**Supplementary File 2**).

## Results

### Study population

Between April 2015 and October 2016, 1,003 adults living with HIV were screened (**Figure S1** in **Supplementary Appendix 1** includes a flowchart of study patients). The final analysis included 995 patients, of which 241 (24.2%) were ART-naïve patients, 671 (67.4%) were ART-experienced patients switching to the new INSTI (ART-switching patients), and 83 (8.3%) were initiating RAL-, EVG/c-, or DTG-based regimens as a part of salvage therapy (ART-salvage patients).

Most patients included in the study were male (n=798, 80.2%). The transmission route was injection drug use in 212 (21.3%) and men who have sex with men (MSM) in 477 (47.9%). At entry, median age was 46 years (IQR: 37–52), median CD4+ T-cell count was 566 cells/μL (IQR: 357–822), and 202 (20.3%) patients had a history of AIDS diagnosis. Baseline characteristics were generally balanced among the three INSTI treatment groups within each ART regimen strategy (**Table 1**). Lamivudine or emtricitabine and abacavir were the most frequently ART backbones used in combination with DTG (604 [94.3%] and 549 [85.8%], respectively) and lamivudine or emtricitabine and both kinds of tenofovir were the most frequently combined with RAL (36 [46.1%] and 35 [45.8%], respectively). ART backbones according to study groups and treatments are detailed in **Table 2**.

**Table 1:** Selected baseline characteristics in ART-naïve patients (n= 241), ART-switching patients (n= 671), and ART-salvage patients (n=83) according to INSTIs started.

	ART-naïve patients (n=241)				ART-switching patients (n=671)				ART-salvage patients (n=83)			
	DTG (n= 150)	EVG/c (n= 80)	RAL (n= 11)	*p-*value	DTG (n= 438)	EVG/c (n= 175)	RAL (n= 58)	*p-*value	DTG (n= 52)	EVG/c (n= 22)	RAL (n= 9)	*p-*value
**Age (years)**, median (IQR)	35 (30.0, 43.0)	33.5 (29.0, 41.5)	42 (28.5, 57.5)	0.056	49 (43.0, 54.0)	45 (38.0, 51.5)	51 (46.0, 54.75)	<0.001	47 (41.0, 53.3)	49.5 (38.0, 57.0)	52.0 (40.0, 52.0)	0.991
**Viral load (copies/mL)**, median (IQR)	53,423 (17,725, 178,801)	38,918 (7,553, 129,512)	88,500 (29,362, 337,493)	0.635	19.00 (19.00, 37.00)	19.00 (19.00, 20.00)	19.00 (19.00, 20.00)	0.814	11,819 (583, 74,013)	2,830 (362, 66,200)	4,420 (1,400, 66,179)	0.007
**Gender**, n (%)				0.930				0.091				0.968
Female	17 (11)	8 (10)	1 (9)		107 (24)	29 (17)	15 (26)		13 (25)	5 (23)	2 (22)	
Male	133 (89)	72 (90)	10 (91)		331(76)	146 (83)	43 (74)		39 (75)	17 (77)	7 (78)	
**Mode of HIV transmission**, n (%)				0.267				<0.001				0.605
MSM	103 (69)	60 (75)	4 (36)		180 (41)	98 (56)	8 (14)		15 (29)	8 (36)	1 (11)	
WSM	31 (20)	15 (19)	4 (36)		113 (26)	44 (25)	15 (26)		16 (31)	5 (23)	3 (33)	
IDU	6 (4)	3 (4)	1 (9)		119 (27)	25 (14)	29 (50)		19 (37)	6 (27)	4 (44)	
Other/unknown	10 (6.7)	2 (2.5)	2 (18.9)		26 (5.9)	8 (4.6)	6 (10.3)		2 (3.8)	3 (13.6)	1 (11)	
**Baseline CD4 count, cells/mm^3^,** median (IQR)	429 (295.25;619.5)	423.5 (267;561.5)	460.5 (105;549.3)	0.535	486 (320)	673.5 (480.25;847.75)	616 (351;821)	0.294	350 (141;659)	435 (202.75;747)	255 (54;432)	0.157
**CD4+ nadir count, cells/mm^3^,** median (IQR)	384.5 (288.3, 551.8)	374 (286, 520)	451 (123, 467)	0.463	222 (98.5, 323.5)	247 (105.5, 391.5)	174 (77.8, 302.5)	0.068	166.5 (71.3, 320.0)	169 (98, 232)	90 (35, 299)	0.971
**HIV stage, n (%)**				0.043				0.648				0.564
A	112 (75)	61 (76)	5 (45)		220 (50)	101 (58)	27 (47)		27 (52)	12 (55)	2 (22)	
B	16 (11)	4 (5)	1 (9)		68 (15)	25 (14)	10 (17)		4 (8)	2 (9)	2 (22)	
C	9 (6)	4 (5)	3 (27)		107 (24)	33 (19)	16 (28)		18 (35)	7 (32)	5 (56)	
Unknown	12 (8)	11 (14)	2 (18)		43 (10)	16 (9)	5 (9)		3 (6)	1 (5)	0 (0)	
**Hepatitis C/B co-infection, n (%)**				0.225				< 0.001				0.822
No	138 (93)	69 (86)	9 (82)		294 (67)	140 (80)	21 (36)		29 (55.7)	17 (77)	5 (56)	
Yes	11 (7.4)	9 (11.3)	2 (18.1)		143 (32.6)	34 (19.4)	2 (18.1)		22 (42.3)	5 (23)	4 (44)	
Unknown	0 (0)	2 (2)	0 (0)		1 (0.2)	1 (0.6)	0 (0)		1 (1.9)	0	0	
**Chronic comorbidities, n (%)**	44 (30)	16 (20)	8 (73)	0.001	268 (61)	80 (46)	43 (74)	< 0.001	27 (52)	11 (50)	4 (44)	0.915
**≥1 genotypic test prior to starting INSTIs, n (%)**				0.019				0.0154				<0.001
Yes	121 (80.7)	51 (63.8)	8 (72.7)		202 (46.1)	98 (56)	21 (36.2)		40 (76.9)	17 (77.3)	6 (66.7)	
**Non-ART treatments, n (%)**					245 (56)	75 (43)	44 (76)	< 0.001	22 (42)	10 (45)	6 (67)	0.399
**Number of previous ART drugs experienced,** median (IQR)					4 (3;7)	3(0;6)	6 (3;9)	<0.001	7 (3;12)	4 (3;10.25)	10 (4.5;12.5)	0.439
**Patients with previous VF,** n (%)					93 (21.2)	24 (13.7)	15 (25.9)	0.0193	23 (44.2)	9 (40.9)	5 (55.6)	0.755

DTG, dolutegravir; EVG/c, elvitegravir plus cobicistat; RAL, raltegravir; MSM, men who have sex with men; WSM, women who have sex with men; IDU, intravenous drug use; INSTIs, integrase strand transfer inhibitors; IQR, interquartile range; VF, virological failure.

**Table 2:** ART-drug backbones according to study groups and treatments.

	DTG	EVG/c	RAL	*p*-value
ART-naïve, *n (%)* n=241	n=150	n=80	n=11	
TDF/FTC	31(20.7)	54 (67.5)	9 (81.8)	<0.001
TAF/FTC	0	26 (32.5)	0	<0.001
ABC/3TC	118 (79.7)	0	1 (9.1)	<0.001
DRV/c	0	0	1 (9.1)	<0.001
RPV	1 (0.7)	0	0	0.737
ART-switching, *n (%)* n=671	n=438	n=175	n=58	
3TC or FTC	414 (94.5)	175 (100)	20 (34.5)	<0.001
TAF or TDF	240 (54.8)	175 (100)	20 (34.5)	<0.001
ABC	396 (90.4)	0	1 (1.7)	<0.001
DRV/c or DRV/r	10 (2.3)	5 (2.8)	3 (5.2)	0.434
ATV unboosted	0	0	1 (1.7)	0.005
RPV	19 (4.3)	0	2 (3.4)	0.020
EFV	0	0	1 (1.7)	0.005
MVC	1 (0.2)	0	0	0.766
ART-salvage, *n (%)* n=83	n=52	n=22	n=9	
3TC or FTC	41 (78.8)	22 (100)	6 (66.7)	0.031
TAF or TDF	9 (17.3)	22 (100)	6 (66.7)	<0.001
ABC	35 (67.3)	0	1 (11.1)	<0.001
DRV/c or DRV/r	12 (23.1)	5 (22.7)	5 (55.6)	0.112
ATV/r	1 (1.9)	0	0	0.739
RPV	2 (3.8)	0	0	0.542
MVC	3 (5.8)	0	1 (11.1)	0.369

3TC, lamivudine; ABC, abacavir; ABC/3TC, abacavir plus lamivudine; ART, antiretroviral treatment; ATV, atazanavir; ATV/r, atazanavir/ritonavir; DTG, dolutegravir; DRV/c, darunavir/cobicistat; DRV/r, darunavir/ritonavir; EFV, efavirenz; EVG/c, elvitegravir plus cobicistat; FTC, emtricitabine; MVC, maraviroc; RAL, raltegravir; RPV, rilpivirine; TDF/FTC, tenofovir disoproxil fumarate plus emtricitabine; TAF, tenofovir alafenamide; TAF/FTC, tenofovir alafenamide plus emtricitabine; TDF, tenofovir disoproxil fumarate; DRV/c, darunavir/cobicistat; RPV, rilpivirine.

### Effectiveness outcomes: virological failure

**Figure 1:**
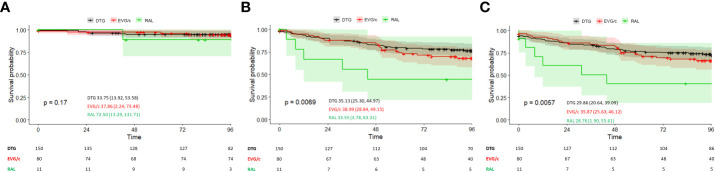
**(A)** Time to virological failure in ART-naïve patients according to INSTI-based regimen (genuine ITT analysis). **(B)** Time to virological failure or switching in ART-naïve patients according to INSTI-based regimen (ITT: S=F sensitivity analysis). **(C)** Time to virological failure or switching in ART-naïve patients or missing during the study according to INSTI-based regimen (ITT: M=F sensitivity analysis). Survival is presented as the mean (95% CI); *p*-values correspond to the log-rank test for intercurve differences. Patients at risk at each timepoint are shown below each graph.

In the ART-naïve group, 150, 80, and 11 patients initiated their first-line regimen with DTG, EVG/c, and RAL, respectively. Based on the primary pre-specified analysis, of 241 ART-naïve patients, 63 (26.1%) reached the composite endpoint of VF or INSTI discontinuation. Of those, 14 (5.8%) patients experienced VF (HIV-1 RNA ≥200 copies/mL) during follow-up and INSTI discontinuation was observed in 49 (77.8%). In comparison with EVG/c and DTG, a limited number of patients initiated RAL as a first-line ART, of whom 18.2% (n=2) experienced VF. Nevertheless, in the genuine ITT analysis, mean time to VF was similar among the three groups (log-rank, *p*=0.17) and between groups on one-to-one comparisons (DTG vs. RAL, *p*=0.082; DTG vs. EVG/c, *p*=0.892; and EVG/c vs. RAL, *p*=0.081) (**Figure 1A**). However, the ITT: S=F sensitivity analysis yielded significant differences. Patients who initiated RAL showed the shortest mean time to failure followed by those initiating DTG and those starting EVG/c (log-rank, *p*=0.007) (**Figure 1B**) (DTG vs. RAL, *p*=0.002 and EVG/c vs. RAL, *p*=0.015). Similar results were observed in the ITT: M=F sensitivity analysis (log-rank, *p*=0.006) (**Figure 1C**), with differences in time to failure between treatments for DTG vs. RAL (*p*=0.002) and EVG/c vs. RAL (*p*=0.007), but not for DTG vs. EVG/c (*p*=0.474).

**Figure 2:**
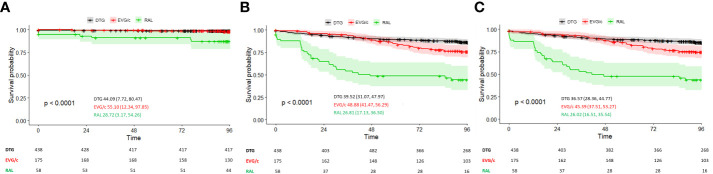
**(A)** Time to virological failure in ART-switching patients according to INSTI-based regimen (genuine ITT analysis). **(B)** Time to virological failure or switching in ART-switching patients according to INSTI-based regimen (ITT: S=F sensitivity analysis). **(C)** Time to virological failure or switching in ART-switching patients or missing during the study according to INSTI-based regimen (ITT: M=F sensitivity analysis). Survival is presented as the mean (95% CI); *p*-values correspond to the log-rank test for intercurve differences. The number of patients at risk at each timepoint are shown below each graph.

In the ART-switching group, 438, 175, and 58 patients initiated DTG-, EVG/c-, and RAL-based regimens, respectively. Out of 671 patients included in this group, 17 (2.5%) experienced VF during follow-up. In addition, INSTIs were discontinued in 117 (17.4%). In the genuine ITT analysis and in the ITT: S=F sensitivity analysis, effectiveness differed among patients who initiated RAL-, EVG/c- or DTG-based regimens (log-rank, *p*<0.001 in both analyses) (**Figures 2A, B**). Time to VF (genuine ITT analysis) differed for DTG vs. RAL (*p*<0.001) and EVG/c vs. RAL (*p*=0.001), but not for DTG vs. EVG/c (*p*=0.827), whereas time to failure in the ITT: S=F sensitivity analysis differed between all treatments: DTG vs. RAL (*p*<0.001), DTG vs. EVG/c (*p*=0.011), and EVG/c vs. RAL (*p*<0.001). Similar results were observed in the ITT: M=F sensitivity analysis (log-rank, *p*<0.001) (**Figure 2C**), where time to failure also differed between all treatments (DTG vs. RAL [*p*<0.001], DTG vs. EVG/c [*p*=0.009], and EVG/c vs. RAL [*p*<0.001]).

**Figure 3:**
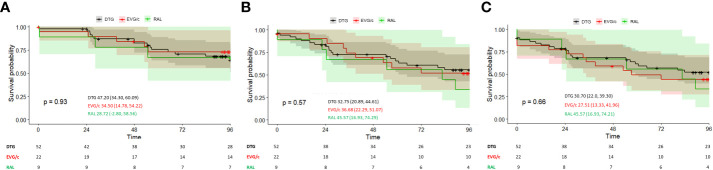
**(A)** Time to virological failure in ART-salvage patients according to INSTI-based regimen (genuine ITT analysis). **(B)** Time to virological failure or switching in ART-salvage patients according to INSTI-based regimen (ITT: S=F sensitivity analysis). **(C)** Time to virological failure or switching in ART-salvage patients or missing during the study according to INSTI-based regimen (ITT: M=F sensitivity analysis). Survival is presented as the mean (95% CI); *p*-values correspond to the log-rank test for intercurve differences. The number of patients at risk at each timepoint are shown below each graph.

Out of the 83 patients included in the ART-salvage group, 52, 22, and 9 patients initiated DTG-, EVG/c-, and RAL-based regimens, respectively. Of 35 (42.1%) patients with the composite endpoint of VF or INSTI discontinuation, 23 (27.7%) experienced VF during follow-up and INSTIs were discontinued in 12 (34.2%). There were no differences between patients who initiated the three INSTI regimens in the genuine ITT analysis (log-rank; *p*=0.93) and in the ITT: S=F sensitivity analysis (log-rank; *p*=0.57) (**Figures 3A, B**). Consistent with these results, one-to-one comparisons lacked statistical significance. Similar results were observed in the ITT: M=F sensitivity analysis (log-rank, *p*=0.66) (**Figure 3C**).

Additional analyses using the clinically valid cut-off for virological failure of VL ≥50 copies/mL are available as supplementary material (**Figures S2–S4**).

### Genotypic resistance analysis

**Table S1** summarises genotyping data for seven patients in the ART-naïve group, one in the ART-switching group, and four in the ART-salvage group.

In the ART-naïve group, one patient had mutations at baseline in the integrase (IN) gene and initiated DTG-based treatment, but had no genotyping data after interrupting treatment voluntarily at week 24. Two other patients with no genotyping data at baseline had mutations at week 24. One patient treated with EVG/c/tenofovir/emtricitabine had mutations in the IN and reverse transcriptase (RT) genes at VF after discontinuing ART. One patient treated with DTG failed to achieve viral suppression at week 24 (HIV-1 RNA: 26,425 copies/mL at week 24 and 8,342 copies/mL at baseline) and had mutations in the protease (PR) and RT genes. The remaining four patients had no genotyping data at baseline or VF, although genotyping was attempted in some of them.

In the ART-switching group, one patient had mutations in the RT gene, detected after VF at week 48. In the ART-salvage group, two patients had mutations at baseline, but no data after experiencing VF at week 48: one patient with a mutation in the IN gene had received EVG/c/emtricitabine/tenofovir, and another patient with mutations in the RT gene had received DTG. One patient with mutations in the PR and RT genes received DTG, and after 48 weeks of poor adherence, additional mutations were found in the IN gene. One patient with no mutations at baseline (IN was not analyzed) received ART with EVG/c/emtricitabine/tenofovir, and after voluntarily interrupting treatment at 24 weeks, the genotyping procedure failed.

One patient in the ART-salvage group had new acquired mutations in the IN gene (E92Q and E157Q) at VF with DTG-based treatment during 48 weeks. In this case, virological resuppression was achieved at week 96 without changes in ART. In the remaining three patients in this group, there were no genotyping tests available.

### Immunological parameters

**Figure 4:**
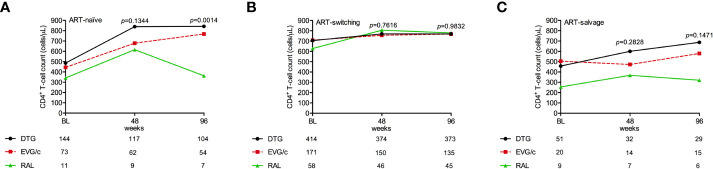
CD4+ T-cell counts in (**A**) ART-naïve, (**B**) ART-switching, and (**C**) ART-salvage groups according to INSTI-based regimen. Data points represent the mean at each time point and error bars represent the standard deviation; the number of patients at each time point for each regimen is shown below each graph. BL, baseline; ns, Not significant.

The median CD4+ T-cell count ( ± SD) in the whole sample increased from 622.2 ± 363.7 cells/μL (n=951) at baseline to 765.8 ± 374.3 (*p*<0.0001) (n=768) at week 96 of follow-up. Differences in CD4+ T-cell count at 96 weeks were not significant in the ART-switching and salvage groups, whilst they reached statistical significance in the ART-naïve group. DTG- and EVG/c-based regimens resulted in greater improvements in the ART-naïve group (**Figure 4**).

### Cox proportional hazard regression models

In ART-naïve patients, a multivariable Cox proportional hazard model showed that CD4+ nadir <100 cells/µL was associated with VF (HR=0.066; 95% CI 0.017–0.257; *p*<0.001) after adjustment for confounding variables (genuine ITT analysis). In both ITT: S=F and ITT: M=F sensitivity analyses, CD4+ nadir <100 cells/µL and RAL- and EVG/c-based regimens were associated with increased risk of VF.

Switching to RAL was associated with a higher risk of VF (HR=8.57; 95% CI 2.995–24.541, *p*<0.001) compared to DTG in the ART-switching group (genuine ITT analysis). However, in the ITT: S=F analysis, both patients who switched to EVG/c (HR=1.66; 95% CI 1.114–2.484; *p*=0.012) and those who switched to RAL (HR=6.16; 95% CI 4.031–9.422; *p*<0.001) had increased HR of VF or INSTI discontinuation. Similarly, these factors were also associated with an increased HR of failure in the M=F sensitivity analysis.

In the ART-salvage group, the HIV-1 RNA >100,000 copies/mL was the only factor associated with VF (HR=3.544; 95% CI 1.352–9.281; *p*<0.010) in the genuine ITT analysis, while we did not find factors associated with failure in the ITT: S=F and ITT: M=F analyses.

### Safety

During the study, 590 AEs were reported in 270 (27.1%) patients, of which 97 (16.4%) were observed in the ART-naïve, 443 (75.1%) in the ART-switching, and 50 (8.5%) in the ART-salvage groups. In addition, 496 (84.1%) AEs were considered non-drug related and 94 (15.9%) were considered drug-related. Of the non-drug related AEs, 79 (81.4%), 375 (84.7%), and 42 (84.0%) were observed in the ART-naïve, ART-switching, and ART-salvage groups, respectively (*p*<0.001). In addition, 18 (18.5%), 68 (15.3%), and 8 (16%) drug-related AEs were observed in the ART-naïve, ART-switching, and ART-salvage groups, respectively (*p*<0.001).

There were no differences observed between DTG, EVG/c, and RAL treatments in drug-related AEs in the ART-naïve (*p*=0.830), ART-switching (*p*=0.945), and ART-salvage (*p*=0.923) groups. Diarrhoea, infections, and psychiatric, musculoskeletal and soft tissue disorders were the most common AEs in the three treatment groups, with no differences between drugs in the three groups (*p*>0.05). AEs leading to discontinuation were also similar for drugs in each group. AEs are summarized in **Table 3**.

**Table 3:** Adverse events according to INSTIs started in ART-naïve patients (n= 241), ART-switching patients (n= 671), and ART-salvage patients (n=83).

	ART-naïve patients				ART-switching patients				ART-salvage patients			
	DTG (n= 150)	EVG/c (n= 80)	RAL (n= 11)	*p*-value	DTG (n= 438)	EVG/c (n= 175)	RAL (n= 58)	*p*-value	DTG (n= 52)	EVG/c (n= 22)	RAL (n= 9)	*p*-value
**Patients experiencing any AE, n (%)**	24 (16.0)	13 (16.3)	2 (18.2)	0.606	119 (27.2)	53 (30.3)	31 (53.4)	<0.001	15 (28.8)	9 (40.9)	4 (44.4)	0.676
**Serious AE, n (%)**	1 (0.7)	2 (2.5)	0 (0.0)	0.665	20 (4.6)	2 (1.1)	17 (29.3)	<0.001	4 (7.7)	3 (13.6)	1 (11.1)	0.652
**Patients experiencing non-drug related AE, n (%)**	17 (11.3)	8 (10.0)	2 (18.2)	<0.001	88 (20.1)	43 (24.6)	27 (46.6)	<0.001	12 (23.1)	7 (31.8)	3 (33.3)	0.838
**Patients experiencing ART-related AE, n (%)**	7 (4.7)	5 (6.3)	0 (0.0)	0.830	31 (7.1)	10 (5.7)	4 (6.9)	0.945	3 (5.7)	2 (9.1)	1 (11.1)	0.923
**Death, n (%)**	0 (0.0)	1 (1.3)	0 (0.0)	0.570	8 (1.8)	0 (0.0)	7 (12.1)	<0.001	2 (3.8)	1 (4.5)	1 (11.1)	0.828
**AIDS event, n (%)**	0 (0.0)	2 (2.5)	0 (0.0)	0.126	4 (0.9)	2 (1.1)	0 (0.0)	0.846	1 (1.9)	1 (4.5)	1 (11.1)	0.586
AE leading to ART discontinuation, n (%)*	3 (.0)	3 (3.8)	0 (0.0)	0.812	17 (3.9)	5 (2.9)	3 (5.2)	0.864	0 (0.0)	1 (4.5)	0 (0.0)	0.422
**AEs (MedRA), n (%)**												
Gastrointestinal disorders	14 (9.3)	9 (11.3)		0.696	36 (8.2)	17 (9.7)	3 (5.2)	0.752	6 (11.5)	3 (13.6)	2 (22.2)	0.857
Infections and infestations	14 (9.3)	2 (2.5)	1 (9.1)	0.289	34 (7.8)	31 (17.7)	15 (25.9)	<0.001	4 (7.7)	7 (31.8)		0.024
Musculoskeletal and connective tissue disorders	8 (5.3)	1 (1.3)	1 (9.1)	0.378	22 (5.0)	22 (12.6)	4 (6.9)	0.013	1 (1.9)	3 (13.6)		0.162
Psychiatric disorders	7 (4.7)	2 (2.5)		0.770	30 (6.8)	8 (4.6)	4 (6.9)	0.765	2 (3.8)	2 (9.1)	2 (22.2)	0.259
Skin and subcutaneous tissue disorders	6 (4.0)	2 (2.5)		0.858	15 (3.4)	11 (6.3)	9 (15.5)	0.001				
Nervous system disorders	4 (2.7)	3 (3.8)	1 (9.1)	0.708	17 (3.9)	2 (1.1)	7 (12.1)	0.003	2 (3.8)	1 (4.5)	1 (11.1)	0.828
Respiratory, thoracic and mediastinal disorders		3 (3.8)		0.106	9 (2.1)	2 (1.1)	7 (12.1)	<0.001	2 (3.8)	1 (4.5)		0.940
Hepatobiliary disorders			2 (18.2)	<0.001								
Eye disorders			1 (9.1)	<0.001								
Endocrine disorders			1 (9.1)	<0.001								
Renal and urinary disorders	2 (1.3)	1 (1.3)	1 (9.1)	0.047								
Metabolism and nutrition disorders					5 (1.1)	7 (4.0)		0.072				
Neoplasms benign, malignant and unspecified (including cysts and polyps)					3 (0.7)	2 (1.1)	5 (8.6)	<0.001	1 (1.9)	2 (9.1)		0.447
Vascular disorders					3 (0.7)	2 (1.1)	5 (8.6)	<0.001				
General disorders and administration site conditions									2 (3.8)	1 (4.5)		0.940
Blood and lymphatic system disorders									1 (1.9)		1 (11.1)	0.321

DTG, dolutegravir; EVG/c, elvitegravir plus cobicistat; RAL, raltegravir; ART, antiretroviral therapy. *Only neuropsychiatric events leading to ART discontinuation were reported.

Overall, 50 serious AEs (grades 4-5) were reported; 2 (66.7%) for EVG/c in the ART-naïve group, and 20 (51.3%) and 4 (50.0%) for DTG in the ART-switching and ART-salvage groups, respectively. Overall, deaths were observed in 1 (0.4%) patient in the ART-naïve group, 15 (2.2%) in the ART-switching group and 4 (4.8%) in the ART-salvage group (**Table 3**).

Laboratory parameters according to treatment in the different study groups throughout the study are summarized in **Tables 3**-**5**. Increases in total cholesterol, HDL-cholesterol, and LDL-cholesterol occurred until 96 weeks of treatment with DTG and EVG/c in ART-naïve patients (**Table 4**). Similar increases were observed in the ART-switching group treated with EVG/c (**Table 5**), while no significant changes were observed in the lipid profile in patients from the ART-salvage group (**Table 6**). Small creatinine increases were also observed in DTG and EVG/c in the ART-naïve and ART-switching groups (**Tables 4**, **5**). Despite being statistically significant, these changes were minimal and within the normal range of the parameters and, therefore, were considered not clinically relevant.

**Table 4:** Laboratory parameters according to INSTIs started in ART-naïve patients.

	Laboratory parameters in ART-naïve patients								
	DTG (n=150)			EVG/c (n=80)			RAL (n=11)		
	Baseline	48 weeks	96 weeks	Baseline	48 weeks	96 weeks	Baseline	48 weeks	96 weeks
LDLc, mean ± SD	95.85 ± 30.23	102.13 ± 29.79	110.34 ± 34.72	98.86 ± 34.03	109.52 ± 32.02	117.31 ± 34.57	81.00 ± 28.48	91.22 ± 30.04	108.86 ± 23.07
*p*-values		*p* = 0.111	*p* = 0.001		*p* = 0.073	*p* = 0.004		*p*= 0.484	*p* = 0.059
HDLc, mean ± SD	41.07 ± 12.94	48.84 ± 13.67	53.99 ± 33.33	39.82 ± 11.09	44.78 ± 15.18	46.62 ± 13.14	38.11 ± 15.62	38.36 ± 11.03	46.04 ± 11.84
*p*-values		*p*< 0.001	*p* = 0.001		*p* = 0.039	*p* = 0.002		*p* = 0.969	*p* = 0.283
TGL, mean ± SD	118.48 ± 93.10	121.82 ± 111.19	116.98 ± 80.25	102.41 ± 62.71	117.05 ± 71.84	130.54 ± 76.97	130.80 ± 90.79	146.40 ± 90.77	150.12 ± 82.95
*p*-values		*p* = 0.7909	*p* = 0.895		*p* = 0.205	*p* = 0.024		*p* = 0.705	*p* = 0.647
TC, mean ± SD	159.05 ± 38.45	172.95 ± 34.24	179.32 ± 30.06	158.28 ± 38.41	174.72 ± 37.09	188.17 ± 43.90	142.50 ± 37.44	164.30 ± 34.05	188.00 ± 31.49
*p*-values		*p*= 0.001	*p* < 0.001		*p* = 0.010	*p* < 0.001		*p* = 0.189	*p* = 0.014
Creatinine, mean ± SD	0.85 ± 0.14	1.00 ± 0.29	0.96 ± 0.19	0.84 ± 0.15	0.94 ± 0.18	0.94 ± 0.16	0.77 ± 0.22	0.96 ± 0.20	0.90 ± 0.26
*p*-values		*p* < 0.001	*p* < 0.001		*p* < 0.001	*p* < 0.001		*p* = 0.050	*p* = 0.270
Kinase, mean ± SD	290.76 ± 696.34	223.64 ± 253.89	186.27 ± 192.36	145.30 ± 122.56	186.77 ± 309.12	168.94 ± 144.30	270.67 ± 444.57	338.20 ± 360.46	225.00 ± 140.01
*p*-values		*p* = 0.488	*p* = 0.321		*p*= 0.567	*p* = 0.548		*p*= 0.820	*p* = 0.901
AST, mean ± SD	35.84 ± 67.04	26.27 ± 13.41	25.38 ± 16.91	35.24 ± 32.20	26.12 ± 14.01	23.50 ± 8.06	31.43 ± 19.85	33.10 ± 15.81	25.62 ± 11.95
*p*-values		*p* = 0.134	*p* = 0.118		*p* = 0.035	*p* = 0.007		*p*= 0.849	*p* = 0.498
ALT, mean ± SD	35.59 ± 73.12	28.46 ± 27.77	26.59 ± 22.12	32.78 ± 27.98	27.93 ± 26.96	23.23 ± 13.00	27.18 ± 19.29	31.00 ± 20.41	25.12 ± 12.04
*p*-values		*p* = 0.300	*p* = 0.211		*p* = 0.289	*p* = 0.014		*p* = 0.664	*p* = 0.794
Glucose, mean ± SD	92.11 ± 23.67	91.23 ± 16.05	90.20 ± 14.04	87.97 ± 7.90	88.07 ± 8.91	89.21 ± 9.54	107.09 ± 46.48	127.40 ± 66.04	116.38 ± 55.96
*p*-values		*p* = 0.723	*p* = 0.451		*p* = 0.943	*p* = 0.409		*p* = 0.421	*p* = 0.697

p-values vs. baseline/DTG, dolutegravir; EVG/c, elvitegravir plus cobicistat; RAL, raltegravir.

DTG, dolutegravir; EVG/c, elvitegravir plus cobicistat; RAL, raltegravir; ART, antiretroviral treatment; ALT, alanine aminotransferase; AST, aspartate aminotransferase; HDLc, high-density lipoprotein cholesterol; LDLc, low-density lipoprotein cholesterol; TC, total cholesterol; TGL, triglycerides; SD, standard deviation.

**Table 5:** Laboratory parameters according to INSTIs started in ART-switching patients.

	Laboratory parameters in ART-switching patients								
	DTG (n=438)			EVG/c (n=175)			RAL (n=58)		
	Baseline	48 weeks	96 weeks	Baseline	48 weeks	96 weeks	Baseline	48 weeks	96 weeks
LDLc, mean ± SD	108.90 ± 35.77	110.69 ± 30.16	111.62 ± 31.44	109.74 ± 31.36	117.54 ± 34.98	119.22 ± 33.59	101.20 ± 38.78	101.31 ± 35.07	95.10 ± 31.87
*p*-values		*p* = 0.475	*p* = 0.288		*p* = 0.053	*p* = 0.019		*p* = 0.988	*p* = 0.412
HDLc, mean ± SD	50.96 ± 21.73	50.19 ± 24.67	49.80 ± 15.02	45.85 ± 13.08	49.68 ± 18.55	49.94 ± 15.15	46.17 ± 15.26	42.46 ± 12.19	41.58 ± 12.42
*p*-values		*p* = 0.656	*p* = 0.415		*p* = 0.044	*p* = 0.018		*p* = 0.209	*p* = 0.118
TGL, mean ± SD	146.89 ± 102.98	136.43 ± 101.23	135.88 ± 108.52	135.04 ± 86.09	141.64 ± 85.19	133.60 ± 77.24	153.60 ± 79.49	142.95 ± 69.61	153.73 ± 81.45
*p*-values		*p* = 0.150	*p* = 0.148		*p* = 0.499	*p* = 0.881		*p* = 0.478	*p* = 0.993
TC, mean ± SD	185.74 ± 42.92	184.77 ± 35.96	186.79 ± 36.95	181.25 ± 36.45	190.68 ± 39.20	192.51 ± 39.28	177.91 ± 45.56	169.37 ± 39.29	165.76 ± 32.47
*p*-values		*p* = 0.731	*p* = 0.715		*p* = 0.027	*p* = 0.010		*p* = 0.315	*p* = 0.133
Creatinine, mean ± SD	0.91 ± 0.23	0.98 ± 0.21	0.98 ± 0.21	0.89 ± 0.19	0.95 ± 0.16	0.93 ± 0.15	0.82 ± 0.30	0.90 ± 0.26	0.90 ± 0.27
*p*-values		*p* < 0.001	*p* < 0.001		*p* = 0.007	*p* = 0.085		*p* = 0.124	*p* = 0.168
Kinase, mean ± SD	138.53 ± 136.52	148.91 ± 120.24	159.63 ± 395.42	146.06 ± 107.90	203.12 ± 354.19	155.21 ± 136.12	92.37 ± 51.29	126.77 ± 89.79	149.77 ± 107.42
*p*-values		*p* = 0.466	*p* = 0.529		*p* = 0.368	*p* = 0.768		*p* = 0.073	*p* = 0.011
AST, mean ± SD	33.00 ± 26.55	30.32 ± 35.91	27.04 ± 14.96	26.52 ± 13.72	29.46 ± 35.16	25.08 ± 14.16	47.22 ± 31.21	26.66 ± 10.87	28.93 ± 12.43
*p*-values		*p* = 0.235	*p* < 0.001		*p* = 0.324	*p* = 0.388		*p* < 0.001	*p* < 0.001
ALT, mean ± SD	37.31 ± 43.46	30.33 ± 31.51	30.02 ± 43.62	29.76 ± 23.02	31.63 ± 30.99	26.26 ± 18.91	54.83 ± 47.68	26.94 ± 18.59	27.63 ± 15.46
*p*-values		*p* = 0.010	*p* = 0.019		*p* = 0.536	*p* = 0.157		*p* < 0.001	*p* < 0.001
Glucose, mean ± SD	94.60 ± 21.99	94.93 ± 20.67	98.24 ± 34.26	94.39 ± 15.57	94.25 ± 14.00	93.92 ± 11.53	107.26 ± 33.80	106.80 ± 32.17	104.38 ± 28.74
*p*-values		*p* = 0.826	*p* = 0.070		*p* = 0.934	*p* = 0.768		*p* = 0.944	*p* = 0.646

p-values vs. baseline/DTG, dolutegravir; EVG/c, elvitegravir plus cobicistat; RAL, raltegravir

DTG, dolutegravir; EVG/c, elvitegravir plus cobicistat; RAL, raltegravir; ART, antiretroviral treatment; ALT, alanine aminotransferase; AST, aspartate aminotransferase; HDLc, high-density lipoprotein cholesterol; LDLc, low-density lipoprotein cholesterol; TC, total cholesterol; TGL, triglycerides; SD, standard deviation.

**Table 6:** Laboratory parameters according to INSTIs started in ART-salvage patients.

	Laboratory parameters in ART-salvage patients								
	DTG (n=52)			EVG/c (n=22)			RAL (n=9)		
	Baseline	48 weeks	96 weeks	Baseline	48 weeks	96 weeks	Baseline	48 weeks	96 weeks
LDLc, mean ± SD	112.69 ± 29.28	121.18 ± 75.24	112.96 ± 43.70	96.47 ± 27.73	98.08 ± 52.42	106.47 ± 39.24	69.40 ± 20.55	96.94 ± 52.25	71.55 ± 13.53
*p*-values		*p* = 0.487	*p* = 0.974		*p* = 0.916	*p* = 0.427		*p* = 0.299	*p* = 0.862
HDLc, mean ± SD	44.77 ± 14.80	46.18 ± 12.64	46.56 ± 13.12	39.80 ± 10.62	43.28 ± 9.51	43.51 ± 11.65	31.20 ± 21.91	37.09 ± 13.63	35.50 ± 16.44
*p*-values		*p* = 0.647	*p* = 0.598		*p* = 0.344	*p* = 0.363		*p* = 0.597	*p* = 0.755
TGL, mean ± SD	132.41 ± 56.87	145.55 ± 60.94	148.37 ± 84.33	117.82 ± 45.53	127.03 ± 72.16	153.40 ± 126.04	127.35 ± 45.72	116.90 ± 68.48	144.73 ± 87.72
*p*-values		*p* = 0.294	*p* = 0.305		*p* = 0.640	*p* = 0.246		*p* = 0.719	*p* = 0.614
TC, mean ± SD	174.11 ± 44.34	186.16 ± 38.52	187.27 ± 45.64	152.44 ± 34.17	166.45 ± 56.09	169.61 ± 43.57	137.98 ± 39.14	158.79 ± 56.31	138.52 ± 44.19
*p*-values		*p* = 0.175	*p* = 0.190		*p* = 0.348	*p* = 0.181		*p* = 0.397	*p* = 0.979
Creatinine, mean ± SD	0.89 ± 0.20	0.98 ± 0.23	0.96 ± 0.22	0.79 ± 0.12	0.90 ± 0.13	0.94 ± 0.12	0.81 ± 0.18	0.91 ± 0.13	0.97 ± 0.23
*p*-values		*p* = 0.049	*p* = 0.119		*p* = 0.007	*p* < 0.001		*p* = 0.256	*p* = 0.144
Kinase, mean ± SD	104.87 ± 106.95	98.45 ± 73.25	101.84 ± 58.02	100.00 ± 44.67	119.21 ± 94.74	127.00 ± 77.32	46.60 ± 19.78	51.76 ± 38.50	66.00 ± 36.77
*p*-values		*p* = 0.822	*p* = 0.912		*p* = 0.645	*p* = 0.459		*p* = 0.800	*p* = 0.381
AST, mean ± SD	37.04 ± 29.12	33.23 ± 28.82	35.91 ± 31.10	38.58 ± 52.04	25.87 ± 13.26	22.75 ± 9.15	37.29 ± 42.23	22.00 ± 18.59	22.50 ± 3.42
*p*-values		*p* = 0.536	*p* = 0.867		*p* = 0.364	*p* = 0.239		*p* = 0.431	*p* = 0.511
ALT, mean ± SD	30.31 ± 23.83	34.25 ± 37.13	31.59 ± 27.37	39.41 ± 55.60	28.53 ± 18.67	20.12 ± 11.29	24.00 ± 36.33	16.86 ± 18.46	11.72 ± 11.11
*p*-values		*p* = 0.541	*p* = 0.821		*p* = 0.445	*p* = 0.168		*p* = 0.643	*p* = 0.405
Glucose, mean ± SD	91.76 ± 12.72	93.69 ± 16.85	95.03 ± 16.90	91.24 ± 12.64	95.43 ± 15.21	93.04 ± 10.03	92.18 ± 12.35	83.57 ± 8.07	85.29 ± 10.03
*p*-values		*p* = 0.533	*p* = 0.309		*p* = 0.359	*p* = 0.635		*p* = 0.133	*p* = 0.250

p-values vs. baseline/DTG, dolutegravir; EVG/c, elvitegravir plus cobicistat; RAL, raltegravir

DTG, dolutegravir; EVG/c, elvitegravir plus cobicistat; RAL, raltegravir; ART, antiretroviral treatment; ALT, alanine aminotransferase; AST, aspartate aminotransferase; HDLc, high-density lipoprotein cholesterol; LDLc, low-density lipoprotein cholesterol; TC, total cholesterol; TGL, triglycerides; SD, standard deviation.

## Discussion

In this multicentre, real-life cohort of HIV-infected patients who initiated INSTIs between April 2015 and October 2016, DTG was always the top performer in all three scenarios (ART-naïve, ART-switching, and ART-salvage patients). However, the virological effectiveness of EVG/c- and RAL-based regimens was similar to that of DTG when given as first-line and salvage therapy. Treatment switches for reasons other than VF were more frequent in patients receiving EVG/c and, in particular, RAL. This likely reflects evolving treatment policies during the study period, when many patients were switched to DTG due to its increased genetic barrier to resistance and convenience. Patients with CD4+ nadir <100 cells/μL (Pisaturo et al., 2021) were more likely to develop VF, mainly if they initiated RAL or EVG/c. In addition, in the ART-switching cohort, initiation of RAL and EVG/c was associated with both VF and INSTI discontinuation. Immunological parameters improved in the three groups and for the three drugs assessed. Treatment safety and tolerability were good and consistent with expected safety profiles, with no new safety signals.

Although few head-to-head comparisons between the three drugs are available (Martinez et al., 2010; Molina et al., 2012; Eron et al., 2013; de Boer et al., 2016; Elzi et al., 2017; Hoffmann et al., 2017; Penafiel et al., 2017; Brehm et al., 2019; Llibre et al., 2019), our findings are consistent with them. In the SPRING-2 study, first-line ART with once-daily DTG had similar antiviral efficacy to twice-daily RAL, in combination with either tenofovir–emtricitabine or abacavir–lamivudine (Raffi et al., 2013a; Raffi et al., 2013b). In addition, a national population-based, prospective, multicentre cohort study in the UK in 4,165 patients with HIV-1 also showed similar antiviral efficacy of ART regimens containing DTG, RAL, or EVG/c in ART-naïve and ART-switching patients.

Safety and tolerability were similar between all three INSTIs in the three scenarios. Reported AEs were consistent with the known safety profiles of the different INSTIs. Diarrhoea, infections, and psychiatric, musculoskeletal, and soft tissue disorders were the most common AEs in all three treatment settings with no differences between drugs (European Medicine Agency - EMA, 2007; European Medicine Agency - EMA, 2013; European Medicine Agency - EMA, 2014; European Medicine Agency - EMA, 2015). This profile of AEs contrasts with previous observations of increased neuropsychiatric side effects in patients treated with DTG, especially amongst women and older patients (de Boer et al., 2016; Menard et al., 2017; Cuzin et al., 2019; Llibre et al., 2019). However, in our study, neuropsychiatric events were not formally investigated using validated neuropsychological assessment tools.

From the limited sample dataset with INSTI genotypic information, emergent INSTI resistance was only observed in two out of 12 patients, one ART-naïve patient with VF to EVG/c and one ART-salvage patient after 48 weeks of poor adherence to treatment with DTG. While EVG/c resistance selection is expected during VF (DeJesus et al., 2012; Sax et al., 2012; Abram et al., 2013), our findings add further evidence that selection of DTG resistance is rare, although possible, in the presence of persistent virus replication (Cahn et al., 2013; Llibre et al., 2019; Revollo et al., 2022).

INSTIs have shown great utility as part of ART regimens in both treatment-naïve and treatment-experienced patients since they were introduced (Brooks et al., 2019). DTG, with a high barrier to resistance, virological potency, superior side-effects profile, lower drug interactions, and lower cost, represented an improvement in previously recommended medications. Its first regulatory approval was in 2013, and, in 2018, WHO recommended it as part of alternative first- and second-line regimens (World Health Organization, 2018; World Health Organization, 2019). Globally, HIV treatment programs have gradually transitioned from non-nucleoside reverse transcriptase inhibitors (NNRTIs), such as efavirenz, to DTG because of its superior efficacy and tolerability and high genetic barrier to HIV drug resistance (Bill and Melinda Gates Foundation, 2020). Projected estimations show how Tenofovir alafenamide and DTG will be major players in the ART regimen by 2025, with 8 million and 15 million patients using these ARVs, respectively (Gupta et al., 2016). Some countries have limited capacity to develop and manage multiple implementation policies, leading to supply shortages and extended lead times (World Health Organization, 2019). In this regard, our findings support that the use of any INSTI is equally effective in any of the usual scenarios of clinical routine, with a view of achieving the objectives of WHO (World Health Organization, 2021). In this sense and despite the fact that DTG is also available in generic formulation in many countries, RAL and EVG/c may still be used as alternative drugs in settings where DTG is not yet available.

Our study has some limitations. Due to the observational nature of this study’s cohort, treatment allocation was not controlled. Thus, the results from this study are potentially affected by several biases, including treatment and channelling biases, precluding control for unmeasured confounders. Moreover, the real-world nature of this study with a consecutive recruitment strategy resulted in the uneven distribution of participants across study groups and treatments, which was small for some groups and treatments (i.e., participants receiving RAL in ART-naïve and ART-salvage groups). Also, BIC was not included in the comparison because it only became widely available towards the end of the study. There were few samples available for genotyping sequencing at failure. A formal neuropsychological evaluation was beyond the scope of this multicentre project; thus, only neuropsychiatric events leading to ART discontinuation were reported. Finally, changes in body weight and composition had not emerged as a concerning complication of INSTI therapy when the cohort was created. Despite these limitations, this study adds to the existing body of real-world evidence of INSTI effectiveness that second-generation INSTIs, such as DTG, are highly efficacious, have a higher genetic barrier to resistance, are well tolerated and convenient, and are generally preferable to treat ART-naïve patients. However, when these drugs are unavailable due to regional or country policies or drug stock-outs in resource-limited settings, first-generation INSTIs achieve high virological effectiveness with a comparable safety profile.

In summary, whereas second-generation INSTIs are preferred treatment options worldwide, and DTG is the treatment of choice in resource-limited settings, first-generation INSTIs may still provide high virological and immunological effectiveness when DTG is, unfortunately, not available.

## INSTINCT study group

IrsiCaixa AIDS Research Institute: MC, MN-J, Mariona Perera. Fight Infections Foundation, Service of Infectious Diseases, Hospital Universitari Germans Trias i Pujol: JRS, RP, Anna Chamorro, Cristina Miranda, Néstor Sánchez, Anna García, Nùria Pérez. University Hospital La Paz: RM-R, Juan González García. Infectious Diseases Unit, Hospital de la Santa Creu i Sant Pau: PD, María del Mar Gutiérrez and María Gracia Mateo. Infectious Diseases Unit, Santiago de Compostela Clinical University Hospital: AA, Elena Losada. Hospital General Universitario Dr. Balmis de Alicante: JP, Sergio Reus, Vicente Boix, Diego Torrús and Esperanza Merino. University Hospital de La Princesa: Jesús Sanz, Ángela Gutiérrez Liarte. Infectious Diseases Unit, La Fe University and Polytechnic Hospital: MM-A. Infectious Diseases Department, Hospital Universitari Vall d’Hebron: JN, Adrià Curran. Infectious Diseases Unit, Elche University General Hospital. MM, Félix Gutiérrez. Infectious Diseases Unit, Complexo Hospitalario Universitario de Ferrol (CHUF): NV-P, Ana Mariño Callejo and Hortensia Álvarez Díaz. HIV Unit, Hospital Álvaro Cunqueiro, Vigo, Spain: Antonio Ocampo Hermida, Celia Miralles, Laura Labajo Leal, Guillermo Pousada. Biomedical Research Center of La Rioja (CIBIR), Logroño, Spain: LP-M, José Ramón Blanco, José Antonio Oteo, Valvanera Ibarra, Mercedes Sanz and Luis Metola. University Hospital Virgen de las Nieves: CG-V, Juan Pasquau. Infectious Diseases Unit, Ramón y Cajal Hospital: MV. HIV and STI Unit, Infectious Diseases Department, Bellvitge University Hospital, Bellvitge Biomedical Research Institute (IDIBELL): AI, Daniel Podzamczer, Camila Piatti. Department of Infectious Diseases, Donostia University Hospital, IIS BioDonostia, Universidad del País Vasco: JI, Maialen Ibarguren, Pilar Carmona-Oyaga. University Hospital San Cecilio: JH-Q. Infectious Diseases Department, Hospital del Mar - IMIM. JV-G. Infectious Diseases Unit, Mataró Hospital: PB, Laia Arbones.

## Data availability statement

The datasets from this study are available upon reasonable request. Requests to access these datasets should be directed to the corresponding author JRS, jrsantos@lluita.org.

## Ethics statement

The study protocol was approved by the local ethics committee of each participating centre, and all patients gave their written informed consent prior to participation. See **Supplementary File 2** for more information. The patients/participants provided their written informed consent to participate in this study.

## Author contributions

JRS and RP designed the INSTINCT study and wrote the manuscript, which was reviewed and approved by all concerning authors. MC performed the laboratory and sequencing data analyses. The remaining authors contributed by collecting clinical data, sampling and Sanger sequencing data (when required) and followed the study subjects. All authors contributed to the article and approved the submitted version.

## Group members of INSTINCT study group

We are grateful to the i2e3 team and especially to Marta Ruiz, MD, PhD, and Sara Cervantes, PhD, for their medical writing assistance, Anna Garcıa, Nestor Sánchez, and Nuria Pérez-Alvarez for their support in the statistical analysis, and Helena Pera and Jessica Toro for their assistance in coordinating and recording all data.
