# Supplementary material for: Effectiveness and safety of integrase strand transfer inhibitors in Spain: a prospective real-world study
Source: Front Cell Infect Microbiol. 2023 Jun 26;13:1187999. doi: 10.3389/fcimb.2023.1187999 (PMC10331300; doi:10.3389/fcimb.2023.1187999)
Supplement: Supplementary file 1 [file DataSheet_1.docx]

# Supplementary file 1

| **Table S1**. Summary of genotyping resistance analysis results. | | | | | |
| --- | --- | --- | --- | --- | --- |
|  | **Baseline genotype** | **ART** | **Virological failure - reason** | **Genotype at virological failure** | **Outcome** |
| **ART-naïve** |  |  |  |  |  |
|  | IN: R263K, T97A; RT: No DRM; PR: No DRM | DTG/abacavir/lamivudine | Week 24 - treatment interruption | Not performed | ART re-initiation and suppression achieved at week 72 |
|  | Not performed | EVG/c/tenofovir disoproxyl fumarate/emtricitabine | Week 24 - treatment interruption | IN: E92Q and E157Q  RT M184V | N/A |
|  | No mutations | DTG/abacavir/lamivudine | Week 24 - treatment interruption | Not performed | N/A |
|  | No mutations | DTG/abacavir/lamivudine | Week 96 – no reason identified | N/A* | Virological failure at week 96 |
|  | No mutations | DTG/abacavir/lamivudine | Week 24 – no reason identified | PR: A71T  RT: No DRM  IN: N/A* | N/A |
|  | Not performed | DTG/abacavir/lamivudine | Week 24 - suppression not achieved | PR: A71T  RT: M184I | Suppression at week 48 |
|  | Not performed | EVG/c/emtricitabine/tenofovir disoproxyl fumarate  Week 24 - switch to RAL/emtricitabine/tenofovir disoproxyl fumarate** | Week 72 - poor adherence | Not performed | Re-suppression at week 96 |
| **ART-switching** | |  |  |  |  |
|  | Not performed | DTG/abacavir/lamivudine | Week 48 – No reason identified | IN: No DRM  RT T69N, K70R, M184V, T215FV and K219Q  PR: No DRM | Re-suppression at week 96 |
| **ART-salvage** | |  |  |  |  |
|  | IN: T97A; RT: No DRM; PR: No DRM | EVG/c/emtricitabine/tenofovir disoproxyl fumarate | Week 48 – No reason identified | N/A* | Week 96: HIV-1 RNA of 1130 copies/mL |
|  | RT: K65R, T69N, V90l, E138A, M184V, M230L  IN: not performed | DTG/abacavir/lamivudine | Week 48 – No reason identified | Not performed | Week 96: HIV-1 RNA of 210 copies/mL. No ART change |
|  | PR: L10I; RT: L74I, K103N, E138A, M184V, P225H, K238T  IN: No DRM | DTG/abacavir/lamivudine +  Darunavir/cobicistat | Week 48 - poor adherence | IN: E92Q and E157Q  PR L10I RT: L74I, K103N, E138A, M184V, P225H, and K238T | Re-suppression at week 96 |
|  | RT: No DRM; PR: No DRM; IN not performed | EVG/c/emtricitabine/tenofovir disoproxyl fumarate | Week 24 - treatment interruption | N/A* | Re-suppression at week 96 |
| DTG, dolutegravir; DRM, drug-related mutations; EVG/c, elvitegravir/cobicistat; IN, integrase gene; N/A, not available; PR, protease gene; RAL, raltegravir; RT, reverse transcriptase gene; ART, antiretroviral treatment.  *Not available due to technical failure of the genotyping procedure (i.e., amplification).  **To avoid potential drug-drug interactions with the treatment for Non-Hodgkin’s lymphoma. | | | | | |


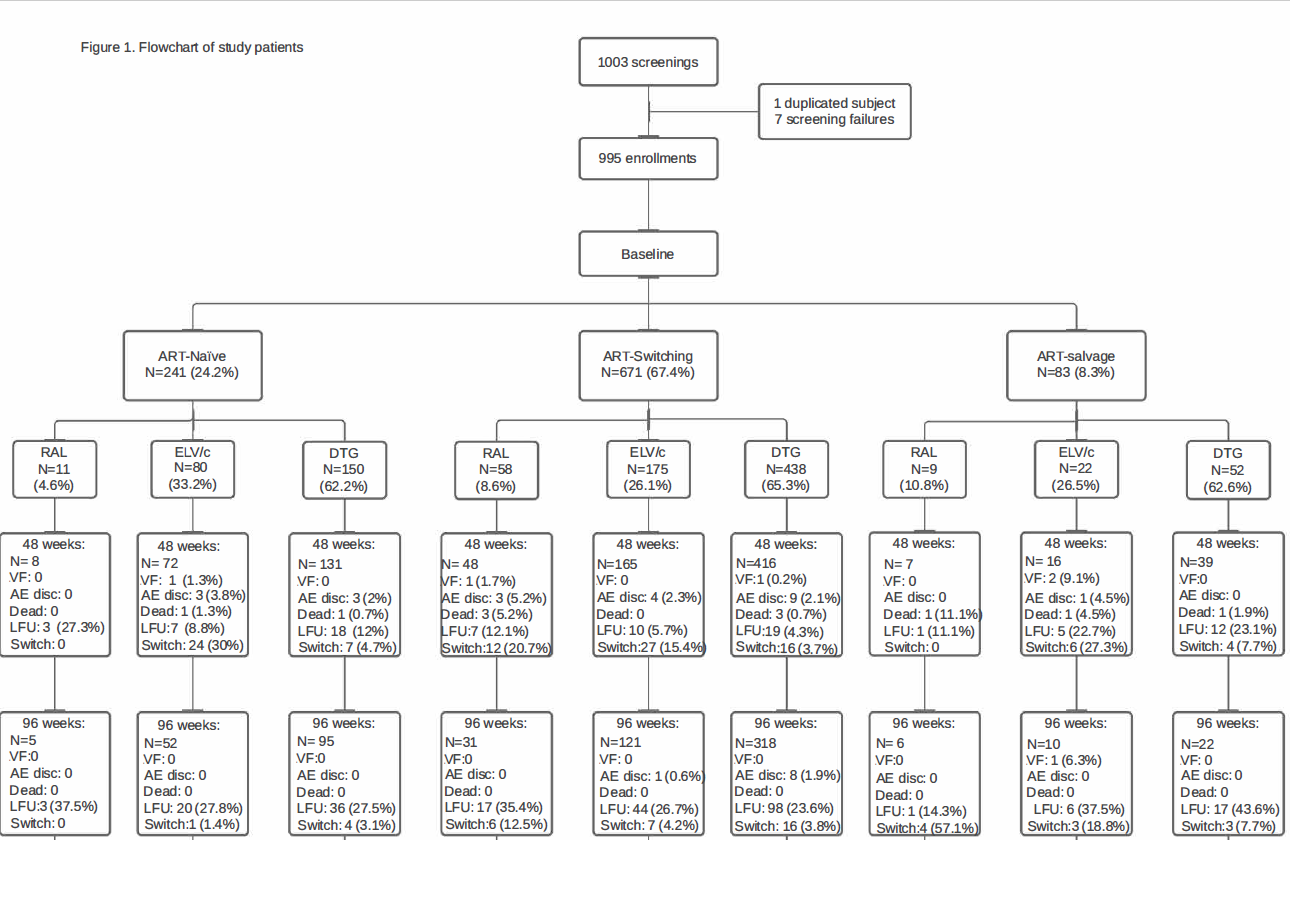
**Figure S1.** Study flow diagram

ART-naïve (ITT sensitivity analysis)

**A. B.**

ART-naïve (ITT analysis)


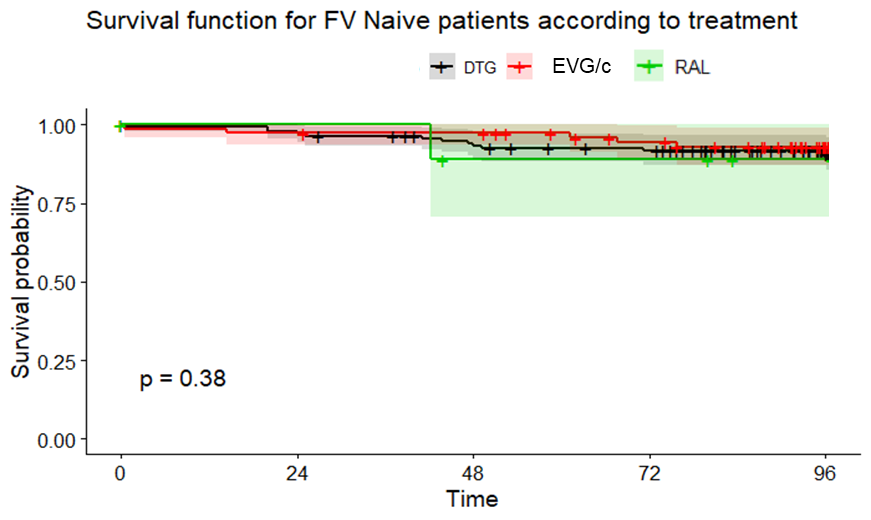

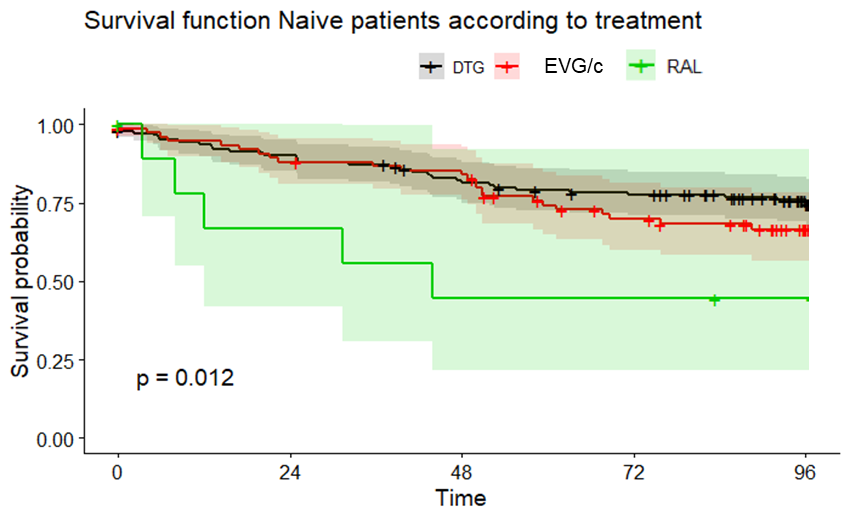


| Patients at risk, n | Baseline | 24 weeks | 48 weeks | 72 weeks | 96 weeks |  | Patients at risk, n | Baseline | 24 weeks | 48 weeks | 72 weeks | 96 weeks |
| --- | --- | --- | --- | --- | --- | --- | --- | --- | --- | --- | --- | --- |
| DTG | 150 | 135 | 125 | 119 | 79 |  | DTG | 150 | 127 | 111 | 103 | 69 |
| EVG/c | 80 | 74 | 68 | 65 | 63 |  | EVG/ | 80 | 67 | 63 | 47 | 39 |
| RAL | 11 | 11 | 9 | 9 | 3 |  | RAL | 11 | 7 | 5 | 5 | 2 |

**Figure S2.** Survival function for virological failure (CV≥50) in ART-naïve patients according to INSTI-based regimen A. ITT analysis. B. ITT sensitivity analysis. The number of patients at risk at each timepoint are shown below each graph.

**A. B.**

ART-switching (ITT sensitivity analysis)

ART-switching (ITT analysis)

| Patients at risk. n | Baseline | 24 weeks | 48 weeks | 72 weeks | 96 weeks |  | Patients at risk, n | Baseline | 24 weeks | 48 weeks | 72 weeks | 96 weeks |
| --- | --- | --- | --- | --- | --- | --- | --- | --- | --- | --- | --- | --- |
| DTG | 438 | 428 | 415 | 403 | 363 |  | DTG | 438 | 403 | 381 | 363 | 264 |
| EVG/c | 175 | 168 | 164 | 157 | 129 |  | EVG/c | 175 | 162 | 146 | 126 | 103 |
| RAL | 58 | 53 | 49 | 44 | 43 |  | RAL | 58 | 37 | 27 | 27 | 16 |

**Figure S3.** Survival function for virological failure (CV≥50) in ART-switching patients according to INSTI-based regimen A. ITT analysis. B. ITT sensitivity analysis. The number of patients at risk at each timepoint are shown below each graph.

ART-salvage (ITT sensitivity analysis)

ART-salvage (ITT analysis)

**A. B.**

| Patients at risk, n | Baseline | 24 weeks | 48 weeks | 72 weeks | 96 weeks |  | Patients at risk, n | Baseline | 24 weeks | 48 weeks | 72 weeks | 96 weeks |
| --- | --- | --- | --- | --- | --- | --- | --- | --- | --- | --- | --- | --- |
| DTG | 52 | 42 | 38 | 29 | 24 |  | DTG | 52 | 38 | 38 | 25 | 22 |
| EVG/c | 22 | 18 | 17 | 13 | 13 |  | EVG/ | 22 | 18 | 14 | 9 | 9 |
| RAL | 9 | 9 | 8 | 7 | 7 |  | RA | 9 | 8 | 7 | 6 | 4 |

**Figure S4.** Survival function for virological failure (CV≥50) in ART-salvage patients according to INSTI-based regimen A. ITT analysis. B. ITT sensitivity analysis. The number of patients at risk at each timepoint are shown below each graph.
