# Supplementary material for: Effectiveness and safety of integrase strand transfer inhibitors in Spain: a prospective real-world study
Source: Front Cell Infect Microbiol. 2023 Jun 26;13:1187999. doi: 10.3389/fcimb.2023.1187999 (PMC10331300; doi:10.3389/fcimb.2023.1187999)
Supplement: Supplementary file 2 [file Table_1.docx]

| **Autonomous Region** | **Hospital** | **Hospital CEIC** | **Approval Date**  **(Hospital CEIC)** | **CEIC Reference** | **Approval Date**  **(Autonomous Region)** | **Reference Opinion Autonomous Region** |
| --- | --- | --- | --- | --- | --- | --- |
| Catalunya | Hospital del Mar | Yes | 24/3/2015 | - | 26/6/2015 | REF: EPA/2015-580/CAT |
|  | Hospital de Mataró | Yes | 25/2/2015 | ref: 08/15 |  |  |
|  | H. Bellvitge | Yes | 5/3/2015 | ref: EPA012/15 |  |  |
|  | H. Vall D'Hebrón | Yes | 27/2/2015 | ref: ID-RTF021 |  |  |
|  | H. Sant Pau | Only Opinion Autonomous Region | - | - |  |  |
|  | H. U. Germans Trias i Pujol | Yes | 10/2/2015 | ref: EPA-15-006 |  |  |
| País Vasco | H. Donosti | Yes | 25/2/2015 | - | 12/3/2015 | REF: EPA2015008 |
| La Rioja | Hospital San Pedro de Logroño | Only Opinion Autonomous Region | - | - | 16/3/2015 | REF: CEICLAR EPA-SP100 |
| Madrid | La Paz | Only Opinion Autonomous Region | - | - | 28/4/2015 | REF: 07/791093.9/15 |
|  | Ramón y Cajal |  |  |  |  |  |
|  | La Princesa |  |  |  |  |  |
| Galicia | H.Clínico U. Santiago de Compostela | Only Opinion Autonomous Region | - | - | 26/2/2015 | REF: 2015/100 |
|  | Xeral de Vigo |  |  |  |  |  |
|  | H. Arquitecto Marcide |  |  |  |  |  |
| Andalucía | H. San Cecilio | Only Opinion Autonomous Region | - | - | 11/5/2015 | REF: 633/4342 |
|  | Virgen de las Nieves |  |  |  |  |  |
| Comunidad Valenciana | H. General U. de Alicante | Only Opinion Autonomous Region | - |  | 26/5/2015 | REF:015/06/02 |
|  | Hospital de la Fe | Yes | 24/3/2015 | FPNT-07-14-EO© |  |  |
|  | Hospital de Elche | Yes | 25/2/2015 | - |  |  |

# Supplementary file 2

CEIC: Clinical Research Ethics Committee
